# Metabolic cost of unloading pedalling in different groups of patients with pulmonary hypertension and volunteers

**DOI:** 10.1038/s41598-024-55980-z

**Published:** 2024-03-05

**Authors:** Till Ittermann, Sabine Kaczmarek, Anne Obst, Raik Könemann, Martin Bahls, Marcus Dörr, Beate Stubbe, Alexander Heine, Dirk Habedank, Ralf Ewert

**Affiliations:** 1 Institute for Community Medicine - SHIP Clinical-Epidemiological Research, University Medicine Greifswald, Walther Rathenau Str. 48, 17475 Greifswald, Germany; 2 German Centre for Cardiovascular Research (DZHK), Partner Site Greifswald, Greifswald, Germany; 3 Department of Internal Medicine B, University Medicine Greifswald, Greifswald, Germany; 4 Department of Internal Medicine, DRK Krankenhaus Berlin, Berlin, Germany

**Keywords:** Biomarkers, Cardiology

## Abstract

Recently, the parameter internal work (IW) has been introduced as change in oxygen uptake (VO_2_) between resting and unloading workload in cardiopulmonary exercise testing (CPET). The proportional IW (PIW) was defined as IW divided by VO_2_ at peak exercise. A second option is to calculate the PIW based on the workload [PIW (Watt)] by considering the aerobic efficiency. The aim of our study was to investigate whether IW and PIW differ between patients with and without pulmonary hypertension and healthy controls. Our study population consisted of 580 patients and 354 healthy controls derived from the Study of Health in Pomerania. The PIW was slightly lower in patients (14.2%) than in healthy controls (14.9%; *p* = 0.030), but the PIW (Watt) was higher in patients (18.0%) than in the healthy controls (15.9%; *p* = 0.001). Such a difference was also observed, when considering only the submaximal workload up to the VAT (19.8% in patients and 15.1% in healthy controls; *p* < 0.001). Since the PIW (Watt) values were higher in patients with pulmonary hypertension, this marker may serve as a useful CPET parameter in clinical practice. In contrast to most of the currently used CPET parameters, the PIW does not require a maximal workload for the patient. Further studies are needed to validate the prognostic significance of the PIW.

## Introduction

Cardiopulmonary exercise testing (CPET) is considered as gold-standard measurement of cardiorespiratory fitness (CRF) and gathers information on cardiac and ventilatory parameters as well as on markers of gas exchange. A reduced CRF is a well-established independent risk factor for cardiovascular morbidity and mortality^1,2^. The inverse relation between CRF and mortality has been reported independent of methods in various study populations^3^. Furthermore, CPET represents an essential method to differentiate indeterminate physiological disturbances, to grade existing disorders, and to monitor therapeutic interventions^4–6^.

Most of the CPET markers currently used in clinical practice require a symptom-limited peak performance of the patient. Peak performance markers have been shown to be predictive for cardiovascular morbidity and mortality^7–9^. However, in the clinical setting a peak performance of the patient is often unfeasible. Therefore, CPET parameters based on workload in the submaximal range but with a similar predictive performance compared to peak CPET markers may be of utmost importance for the real world situation. First approaches of such a marker based on oxygen uptake kinetics were introduced in 1992^10^. More recently the oxygen uptake efficiency slope has been described as an effective measure of pathological exercise physiology^11^. Likewise, the cardiorespiratory optimal point has been presented as submaximal CPET marker highly predictive for mortality^12^.

Recently, a further parameter based on the metabolic cost of exercise unloading pedalling also known as internal work (IW) has been reported^13^. This marker and its derivatives are based on the workload, which occurs when changing from the resting to the unloading phase in the CPET. In the Framingham Heart Study (FHS), IW was positively associated with cardiovascular risk factors in healthy subjects as well as in heart failure patients with preserved Ejection Fraction (HFpEF)^13^. In a recent analysis from our group a higher proportional IW (PIW = IW divided through peak oxygen uptake [VO_2_]) was associated with a higher all-cause mortality in healthy individuals of the population-based Study of Health Pomerania^14^. However, it is so far unclear whether IW and PIW are good markers to differentiate other patient groups than HFpEF from healthy individuals. Therefore, the aim of our study was to investigate whether IW and PIW differ between several patient groups with and without pulmonary hypertension and healthy controls.

## Methods

### Patients

Patients with and without validated pulmonary hypertension (defined as mean pulmonary arterial pressure [PAPmean] > 20 mmHg and pulmonary vascular resistance [PVR] > 2 WU^15^) were enrolled at the Department of Internal Medicine B of the University Medicine Greifswald between 2010 and 2022 based on a right heart catheter database. The four patient groups with pulmonary hypertension comprised patients with chronic thromboembolic pulmonary hypertension (CTEPH), patients with HFpEF, patients with idiopathic pulmonary artery hypertension (IPAH) and patients with pulmonary arterial hypertension in connective tissue disorders (PAH-CTD). The patients without validated pulmonary hypertension were defined as patient controls. All patients gave informed written consent, the study followed the principles of the Declaration of Helsinki and was approved by the Ethics Committee of the University Medicine Greifswald (approval numbers BB 216/20, BB 057/17 and BB 167/18).

From the 818 patients enrolled in the study we excluded 80 patients without a CPET measurement, 50 patients diagnosed as pulmonary hypertensive but with a PAPmean ≤ 20 mmHg, 73 patient controls with a PAPmean > 20, 12 patients with a respiratory exchange ratio < 1.05, and 23 patients with invalid VO_2_ values at rest (< 2 ml/min/kg) or at unload (< 3 ml/min/kg) resulting in a patient population of 580 individuals [470 with validated pulmonary hypertension and 110 patient controls (Supplementary Fig. 1)].

### Healthy controls

The SHIP project includes several large population-based studies, which were all conducted in the Northeast of Germany. In the first SHIP cohort; SHIP-START, 6265 individuals aged 20–79 years were selected from population registries, of which 4308 individuals (response 68.8%) participated between 1997 and 2001^16^. In the present analyses we used data from the second follow-up (SHIP-START-2), in which 2333 individuals aged 30–93 years were examined between 2008 and 2012. SHIP was conducted according to the guidelines of the Declaration of Helsinki and an informed written consent was provided by each participant. SHIP was approved by the Ethics Committee of the University Medicine Greifswald (approval number BB 39/08). In SHIP-START-2 all CPET parameters considered in this manuscript were available for 461 individuals (Supplementary Fig. 1). Furthermore, we excluded 89 individuals with history of cancer, atrial fibrillation, coronary heart disease, COPD, or an obstructive ventilator defect, and 18 individuals with invalid VO_2_ measurements resulting in a population of 354 healthy controls.

### Assessments

The calculation of lung function parameters was performed according to the normal values as described before^17,18^. Obstructive pulmonary disease was defined by forced expiratory volume in 1 s (FEV1)/forced vital capacity (FVC) < 70%; restrictive pulmonary disease by total lung capacity (TLC) < 80%; and clinically relevant diffusion impairment by diffusion capacity of carbon monoxide (DLCO) < 60% of normal. The Krogh Index was defined as the DLCO corrected for alveolar volume (KCO). Diffusion values were corrected for hemoglobin (DLCOc/KCOc).

**Figure 1:**
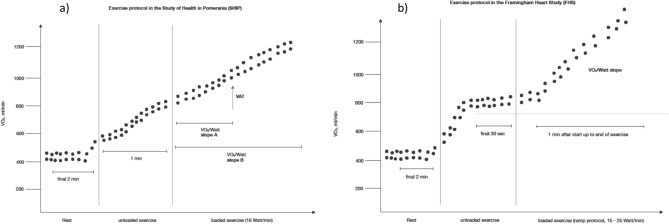
Study protocols of cardiopulmonary exercise testing in (**a**) our study and in (**b**) the Framingham heart study.

In both the patients and the healthy controls a symptom-limited exercise test using a calibrated electromagnetically braked cycle ergometer (Ergoselect 100, Ergoline, Germany) was performed according to a modified Jones protocol including a 3-min resting period, a 1-min unloaded cycling following with an increase of power by 16 W each minute up to exhaustion (Fig. 1a). The participants were instructed to keep a pedaling cadence of 50 to 60 per minute. The criteria for a maximal exercise was a RER > 1.05, maximal metabolic workload or ventilatory limitation (dynamic hyperinflation of the lung characterized by a Vt/IC > 0.75). VO_2_ was analyzed breath-by-breath averaged over 10-s intervals using the Oxycon Pro system (Jaeger/Viasys Healthcare; Hoechberg, Germany), together with a Rudolph’s mask, which was recalibrated before each test. VO_2_@rest was defined as the median VO2 value during the final 2-min resting period. VO_2_peak in ml/min was defined as the highest 10-s average of absolute VO_2_ during late exercise or early recovery^19,20^. Predicted VO_2_peak values in % were calculated according to the age-, sex-, height- and weight-specific normal values^19^. A reduced cardiopulmonary exercise capacity was defined as VO_2_peak values below 60% of predicted values as this cut-off has been shown to be predictive for mortality in patient groups^21–24^.

We defined the IW (Watt) according to the first description by Shah et al.^13^. This parameter was calculated by dividing the difference between VO_2_@rest and VO_2_ at unloading exercise by the aerobic efficiency (VO_2_/Watt slope) calculated during loaded exercise. Since the CPET protocols differed slightly between our study and the Framingham Heart Study, we visualized both protocols in Fig. 1. We calculated aerobic efficiency using VO_2_ and workload after initiation of incremental ramp exercise to ventilatory anaerobic threshold (VAT) and to peak exercise (Fig. 1). The VO_2_ uptake at rest was defined as the median value of the VO_2_ displayed in in the last 2 min of the resting period (steady state) of the CPET. The VO_2_ uptake of unloaded exercise was defined as the median value of the VO_2_ displayed in the whole time of 60 s of the unloaded exercise period (Fig. 1). Furthermore, we calculated the simple IW (ml/min) as difference of VO_2_@unload and VO_2_@rest^14^.

According to Shah et al. we calculated the PIW (IW(Watt)/Watt_max_ in %) as the ratio of IW (Watt) to the total work (in Watt)^13^. Additionally, we calculated the simple PIW by the following formula: ((VO_2_@unloading − VO_2_@rest)/VO_2_@peak)*100^14^.

### Statistical methods

Stratified by patient groups and controls, continuous data are reported as median, 25th and 75th percentile and categorical data as percentages. Difference among groups were evaluated by Mann–Whitney U (continuous data, two groups), Kruskal Wallis (continuous data; more than two groups) and χ2-tests (categorical data). We conducted linear regression models adjusted for age, sex, and BMI to evaluate differences in CPET markers between the groups. Furthermore, linear regression models adjusted for age and sex were used to associate metabolic and functional parameters with IW and PIW. We considered a *p* < 0.05 as statistically significant. Stata 18.0 was used for analyses (Stata Corporation, College Station, TX, USA).

## Results

### Description of the study population

The SHIP controls were in median younger than all patient groups (Table 1). In the patient groups individuals with IPAH had the lowest median age. The percentage of females was highest in the PAH-CTD group and lowest in the CTEPH group. The median BMI was highest in the HFpEF group and lowest in the PAH-CTD group. Percentage of current smokers was lowest in the SHIP controls and the PAH-CTD group and highest in the patient controls. The CTEPH group comprised the highest percentage of venous thromboembolism, while the prevalence for type 2 diabetes, arterial hypertension, chronic kidney failure, coronary heart disease, and COPD was highest in the HFpEF group. Cancer was most frequently observed in the patient controls, while peripheral artery disease and interstitial lung disease was most prevalent in the PAH-CTD group. Diastolic dysfunction and verified tricuspid valve insufficiency were most frequently observed in the HFpEF and IPAH group. The IPAH group had the highest median values of PAPmean and PVR. Median lung function parameters were most unfavorable in the HFpEF group, whereas median diffusion markers were lowest in the IPAH and PAH-CTD groups. The IPAH group also had the lowest maximal workload (as % predicted) as well as the lowest median VO_2_ values at the ventilatory anaerobic threshold and at peak, closely followed by the PAH-CTD group. Likewise, the anaerobic capacity (V0_2_/Watt at VAT and peak) was lowest in IPAH group.

**Table 1:** Characteristics of the study population stratified by groups of patients and healthy controls.

	CTEPH (n = 106)	HFpEF (n = 175)	IPAH (n = 88)	PAH-CTD (n = 101)	Patient controls (n = 110)	SHIP controls (n = 354)	p1*	p2*
Age (years)	66 (56; 77)	73 (66; 77)	59 (46; 70)	67 (59; 74)	66 (55; 74)	55 (45;63)	< 0.001	< 0.001
Females	39.6	54.3	53.4	75.3	56.4	50.3	< 0.001	0.120
Height (cm)	173 (166; 179)	167 (160; 174)	170 (162; 178)	166 (160; 172)	168 (163; 175)	170 (163;177)	< 0.001	0.008
Weight (kg)	85 (75; 96)	84 (72; 95)	80 (62; 92)	70 (63; 82)	77 (66; 94)	79.3 (70.6;90.4)	< 0.001	0.705
Body mass index (kg/m^2^)	27.9 (25.3; 31.6)	29.8 (26.0; 34.7)	27.5 (23.2; 30.7)	25.5 (23.0; 28.9)	27.7 (24.2; 31.0)	27.0 (24.8;30.2)	< 0.001	0.056
Body surface area (m^2^. Dubois)	1.98 (1.84; 2.15)	1.93 (1.77; 2.05)	1.89 (1.70; 2.07)	1.79 (1.69; 1.95)	1.89 (1.72; 2.06)	1.92 (1.79;2.06)	< 0.001	0.472
Current smoker (%)	19.8	25.1	26.1	16.8	29.1	17.0	0.215	0.016
Type 2 diabetes (%)	17.9	39.4	19.3	13.9	17.3	8.2	< 0.001	< 0.001
Arterial hypertension (%)	53.8	76.0	64.8	70.3	45.5	54.0	< 0.001	0.004
Atrial fibrillation (%)	11.3	57.7	14.8	15.8	11.8	0.0%	< 0.001	< 0.001
Peripheral artery disease (%)	2.8	4.6	2.3	5.9	3.6	–	0.689	–
Chronic kidney failure (%)	17.0	29.1	14.8	11.9	8.2	2.0	< 0.001	< 0.001
Cancer (%)	17.9	16.0	11.4	13.9	19.1	0.0	0.585	< 0.001
Coronary heart disease (%)	17.0	39.4	26.1	18.8	25.5	0.0	< 0.001	< 0.001
COPD/Asthma (%)	17.0	27.4	15.9	5.9	18.2	0.0	< 0.001	< 0.001
Venous thromboembolism (%)	68.9	14.3	13.6	9.9	36.4	–	< 0.001	–
Cerebrovascular disease (%)	6.6	3.4	3.4	5.9	2.7	–	0.529	–
Interstitial lung disease (%)	17.9	18.3	18.2	82.2	22.7	–	< 0.001	–
Echocardiography								–
Diastolic dysfunction (%)	20.8	40.0	40.9	33.7	19.1	–	< 0.001	–
TAPSE	20 (15; 24)	20 (16; 23)	19 (16; 22)	20 (17; 24)	23 (21; 26)	23 (21; 26)	< 0.001	< 0.001
Verified tricuspid valve insufficiency (%)	49.1	62.3	59.1	56.4	30.9	–	< 0.001	–
Estimated systolic PAP	63 (52; 76)	46 (36; 62)	71 (51; 86)	46 (35; 65)	38 (31; 44)	–	< 0.001	–
Right heart catheter								–
Mean right atrial pressure (mmHg)	7 (5; 11)	10 (7; 16)	8 (5; 11)	6 (3; 9)	5 (2; 8)	–	< 0.001	–
PAPmean (mmHg)	38 (29; 51)	32 (26; 43)	52 (43; 59)	30 (19; 45)	17 (14; 19)	–	< 0.001	–
PAWP (mmHg)	12 (9; 14)	19 (15; 26)	12 (9; 14)	11 (7; 15)	9 (7; 13)	–	< 0.001	–
PVR; WU	5.6 (2.9; 8.7)	2.7 (1.7; 3.9)	8.0 (5.7; 11.1)	3.7 (1.7; 7.0)	1.5 (0.9; 2.2)	–	< 0.001	–
Cardiac output (C0); l/min	4.8 (4.0; 5.8)	4.8 (4.0; 5.7)	4.9 (3.9; 5.8)	4.8 (4.0; 5.8))	5.0 (4.2; 5.9)	–	0.845	–
Lung function								
Total lung capacity (TLC) (% predicted)	98 (86; 106)	88 (76; 99)	99 (87; 109)	90 (74; 100)	98 (83; 108)	100 (93; 110)	< 0.001	< 0.001
Vital capacity (VC) (% predicted)	81.0 (71.4; 93.5)	76.6 (60.0; 86.1)	87.3 (75.7; 101)	84.1 (64.6; 88.9)	84.9 (67.1; 99.8)	105.6 (95.4; 120.2)	0.001	< 0.001
Forced vital capacity (FVC) (% predicted)	87.3 (70.6; 102)	78.0 (63.6; 93.0)	92.9 (76.4; 106)	87.0 (73.2; 95.1)	88.4 (73.1; 104)	108.3 (97.9; 120.5)	< 0.001	< 0.001
FEV1 (% predicted)	78.8 (66.2; 94.4)	73.4 (61.3; 87.5)	81.1 (67.9; 94.3)	82.2 (69.2; 94.3)	85.2 (65.6; 103)	100.5 (92.0; 107.7)	0.017	< 0.001
FEV1/FVC (%)	73 (69; 80)	75 (69; 81)	75 (69; 79)	79 (73; 85)	79 (72; 83)	80.5 (77.6; 83.2)	0.007	< 0.001
Residual volume (RV) (% predicted)	113 (99; 135)	110 (90; 129)	122 (103; 140)	105 (79; 127)	113 (100; 131)	99 (78; 117)	0.009	< 0.001
RV/TLC (%)	45.7 (40.6; 53.4)	52.3 (45.1; 58.1)	42.6 (36.7; 50.3)	45.4 (39.5; 54.8)	47.0 (40.3; 55.5)	35.0 (28.0; 41.0)	< 0.001	< 0.001
DLCOc (% predicted)	58.1 (45.9; 70.3)	53.2 (38.7; 68.1)	48.4 (34.9; 59.7)	39.5 (32.0; 62.0)	62.8 (43.1; 76.0)	99.9 (92.5; 107.1)	0.002	< 0.001
KCOc (% predicted)	72.4 (59.6; 83.0)	76.3 (53.1; 88.4)	58.9 (45.1; 70.0)	58.2 (43.3; 78.1)	73.7 (54.3; 90.8)	100.8 (93.2; 110.6)	< 0.001	< 0.001
CPET								
Maximal workload (Watt)	84 (68; 116)	68 (68; 91)	84 (60; 100)	68 (52; 84)	84 (68; 100)	164 (132; 196)	< 0.001	< 0.001
Maximal workload (% predicted)	68.7 (47.8; 96.2)	78.5 (52.9; 104)	61.6 (50.1; 88.9)	83.1 (61.7; 106)	84.9 (61.0; 113)	123 (102; 150)	< 0.001	< 0.001
Heart rate (at rest)	78 (70; 90)	70 (62; 81)	77 (68; 89)	79 (70; 93)	75 (68; 88)	76 (67; 85)	< 0.001	0.903
Heart rate (maximal)	118 (101; 137)	102 (88; 116)	119 (100; 140)	115 (98; 133)	110 (99; 133)	157 (142; 171)	< 0.001	< 0.001
VO_2_ (ml/min) at rest	353 (294; 416)	340 (289; 396)	332 (276; 388)	319 (272; 372)	308 (261; 383)	283 (231; 335)	0.001	< 0.001
VO_2_ (ml/min) at unloading exercise	510 (432; 613)	496 (414; 557)	479 (404; 535)	449 (397; 532)	498 (412; 618)	580 (487; 670)	0.007	< 0.001
VO_2_ (ml/min) at VAT	802 (664; 975)	763 (632; 920)	659 (548; 850)	677 (541; 805)	812 (660; 977)	950 (800; 1200)	< 0.001	< 0.001
VO_2_ (ml/min) at peak	1122 (881; 1461)	1066 (870; 1278)	951 (726; 1205)	927 (703; 1179)	1206 (945; 1489)	1900 (1533; 2432)	< 0.001	< 0.001
V02 peak (% predicted)	59.9 (46.4; 71.8)	63.2 (53.4; 74.1)	51.4 (40.5; 64.0)	58.6 (43.6; 74.0)	69.5 (55.5; 79.8)	98.6 (86.8; 109.9)	< 0.001	< 0.001
V0_2_/Watt (ml/Watt) at peak	9.4 (8.0; 11.7)	11.6 (9.3; 16.1)	8.7 (6.9; 10.4)	9.5 (6.2; 11.7)	10.8 (9.4; 13.8)	11.5 (10.3; 12.8)	< 0.001	< 0.001
V0_2_/Watt (ml/Watt) at VAT	9.2 (7.2; 12.0)	9.9 (8.2; 12.6)	7.9 (5.7; 10.2)	8.8 (5.8; 11.2)	10.1 (8.6; 11.9)	12.1 (10.1;14.8)	< 0.001	< 0.001
Calculated parameters								
Internal work (IW) (ml/min)	146 (96; 223)	149 (104; 199)	144 (100; 181)	128 (91; 189)	189 (131; 242)	293 (226; 353)	< 0.001	< 0.001
Proportional IW (%)	13.0 (10.4; 16.6)	13.5 (10.0; 16.9)	15.3 (11.1; 19.5)	14.1 (10.2; 18.1)	15.2 (12.5; 18.6)	14.9 (11.8; 18.4)	0.018	0.030
IW (Watt)	16.1 (9.7; 21.3)	12.1 (6.7; 16.4)	16.4 (10.0; 22.4)	13.5 (7.9; 22.5)	16.9 (10.3; 23.5)	25.3 (19.1; 31.5)	< 0.001	< 0.001
Proportional IW (IW/Watt_max_ in %)	18.3 (13.2; 23.1)	14.9 (9.6; 22.7)	20.9 (13.2; 28.9)	18.3 (10.7; 27.3)	18.6 (11.7; 25.0)	15.9 (11.4; 20.0)	0.004	0.001
IW@VAT (Watt)	16.7 (12.7; 21.4)	15.0 (12.0; 19.2)	17.8 (14.5; 24.0)	17.7 (11.6; 23.7)	18.0 (13.8; 24.3)	23.5 (18.3; 30.8)	< 0.001	< 0.001
Proportional IW@VAT (IW/Watt_max_ in %)	18.4 (14.4; 23.6)	18.8 (14.0; 27.3)	21.7 (17.2; 33.4)	20.9 (15.2; 32.8)	19.9 (15.2; 25.4)	15.1 (11.7; 19.2)	0.024	< 0.001

Continuous data are reported as median. 25th and 75th percentile; categorical data as percentage.

p1 *p*-values between patient groups (Kruskal Wallis test for continuous data; χ^2^-test for categorical data).

p2 *p*-value between patients and SHIP controls (Mann–Whitney-U test for continuous data; χ^2^-test for categorical data).

CTEPH chronic thromboembolic pulmonary hypertension; HFpEF heart failure with preserved ejection fraction; IPAH Idiopathic pulmonary arterial hypertension; PAH-CTD pulmonary arterial hypertension with connective tissue disease.

PAPmean, Mean pulmonary artery pressure; PAWP, Pulmonary artery wedge pressure; PVR, Pulmonary vascular resistance; FEV1, Forced expiratory volume in 1 s; DLCOc, Diffusing capacity of the lungs for carbon monoxide corrected to hemoglobin value; KCOc, Transfer coefficient of the lung for carbon monoxide corrected to hemoglobin value; VAT, Ventilatory anaerobic threshold.

The patient groups had significantly lower median IW (ml/min and Watt) values than the SHIP controls (Table 1). The median PIW values were comparable between the patients (14.2%) and the SHIP controls (14.9%) and ranged from 13.0% in the CTEPH group to 15.2% in the patient controls. The overall PIW in the patients was 14.2% and 14.9% in the SHIP controls. The median IW (Watt) was highest in the patient controls followed by the IPAH and the CTEPH group. The median PIW (Watt) was highest among the IPAH group followed by the patient controls. In all patients, the median PIW (Watt) was 18.0%, while in the SHIP controls the median PIW (Watt) was 15.9%. The additionally calculated PIW considering the values up to the VAT revealed a comparable distribution (19.8% in patients and 15.1% in SHIP controls).

### Multivariable analyses

Adjusted for age, sex, and BMI, all patient groups had a significantly higher mean VO_2_@ rest as well as a lower VO_2_@unload and VO_2_@peak compared to the SHIP controls (Table 2). Likewise, the IW was significantly lower in all patient groups compared to the SHIP controls and the CTEPH, PAH-CTD and HFpEF patients had a significantly lower PIW than the SHIP controls. The IW (Watt) was significantly lower in all patient groups compared to the SHIP controls, while the PIW (Watt) was significantly higher in the IPAH and PAH-CTD groups compared to the SHIP controls.

**Table 2:** Associations of the groups with markers of spiroergometry compared to SHIP controls.

	CTEPH β (95%-CI)	HFpEF β (95%-CI)	IPAH β (95%-CI)	PAH-CTD β (95%-CI)	Patient controls β (95%-CI)	SHIP controls
VO_2_ (ml/min) at rest	67.2 (53.0; 81.4)*	62.1 (49.2; 75.1)*	53.9 (39.1; 68.7)*	73.1 (58.4; 87.8)*	43.7 (29.8; 57.6)*	Reference
VO_2_ (ml/min) at unloading exercise	− 56.0 (− 77.2; − 34.7)*	− 58.6 (− 78.0; − 39.2)*	− 84.6 (− 107; − 62.5)*	− 49.7 (− 71.7; − 27.7)*	− 44.4 (− 65.3; − 23.6)*	Reference
VO_2_ (ml/min) at peak	− 760 (− 848; − 672)*	− 688 (− 769; − 608)*	− 985 (− 1077; − 893)*	− 737 (− 828; − 645)*	− 594 (− 681; − 508)*	Reference
Internal work (IW) (ml/min)	− 121 (− 138; − 104)*	− 119 (− 135; − 103)*	− 136 (− 154; − 118)*	− 121 (− 139; − 104)*	− 86.3 (− 103; − 69.5)*	Reference
Proportional IW (%)	− 1.92 (− 3.09; − 0.75)*	− 1.98 (− 3.05; − 0.91)*	0.02 (− 1.20; 1.23)	− 1.61 (− 2.82; − 0.39)*	− 0.25 (− 1.39; 0.90)	Reference
Internal work (IW) (Watt)	− 8.31 (− 10.3; − 6.35)*	− 10.5 (− 12.3; − 8.70)*	− 7.78 (− 9.81; − 5.74)*	− 6.81 (− 8.84; − 4.78)*	− 7.10 (− 9.02; − 5.18)*	Reference
Proportional IW (IW/Watt_max_ in %)	1.08 (− 1.06; 3.22)	− 0.85 (− 2.80; 1.11)	5.77 (3.55; 8.00)*	3.70 (1.47; 5.93)*	1.92 (− 0.16; 4.02)	Reference

Coefficients are derived from linear regression models adjusted for age, sex, and body mass index.

CI confidence interval; *p < 0.05.

**Figure 2:**
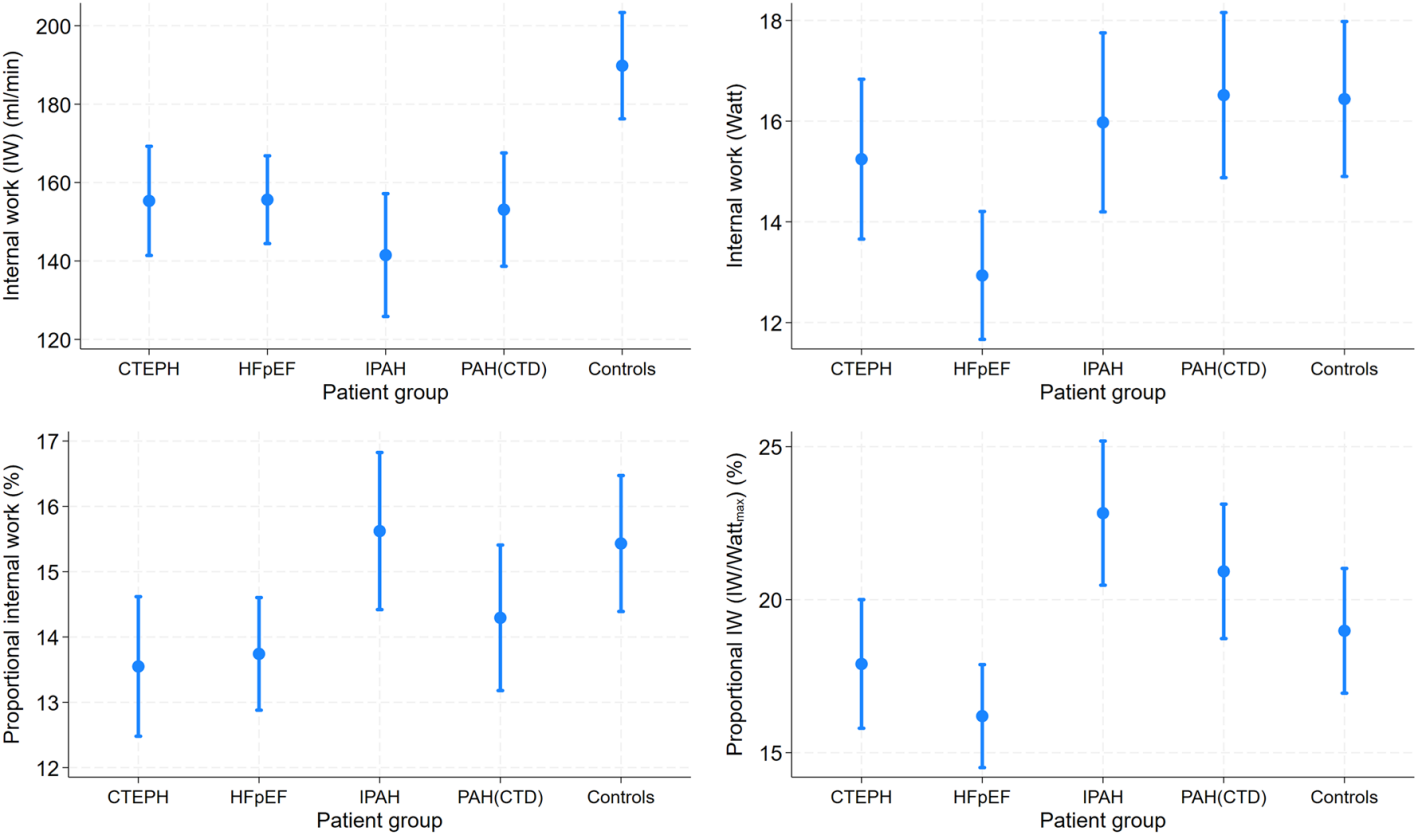
Mean differences in internal work and proportional internal work among the patient groups adjusted for age, sex, and body mass index.

Compared to the patient controls (Table 3, Fig. 2), the CTEPH group had a significantly higher VO_2_@rest as well as a significantly lower VO_2_@peak, IW (mL/min) and PIW. The HFpEF patients had significantly higher V0_2_@rest values and lower values of V0_2_@peak, IW (ml/min and Watt) and PIW than the patient controls. The IPAH patients had significantly lower values of VO_2_@unload, VO_2_@peak, IW (ml/min), and PIW (Watt) than the patient controls. The PAH-CTD patients had a significantly higher VO_2_@rest compared to the patient controls, but lower values of VO_2_@peak, and IW (mL/min).

**Table 3:** Associations of the groups with markers of spiroergometry compared to patient controls.

	CTEPH β (95%-CI)	HFpEF β (95%-CI)	IPAH β (95%-CI)	PAH-CTD β (95%-CI)	Patient controls β (95%-CI)
VO_2_ (ml/min) at rest	24.3 (6.80; 41.7)*	18.3 (2.35; 34.3)*	10.6 (− 7.88; 29.1)	28.3 (10.5; 46.1)*	Reference
VO_2_ (ml/min) at unloading exercise	− 10.2 (− 34.0; 13.6)	− 15.9 (− 37.6; 5.88)	− 37.7 (− 62.9; − 12.6)*	− 8.40 (− 32.6; 15.8)	Reference
VO_2_ (ml/min) at peak	− 131 (− 219; − 42)*	− 122 (− 203; − 41)*	− 357 (− 451; − 263)*	− 193 (− 284; − 103)*	Reference
Internal Work (IW) (ml/min)	− 34.5 (− 53.9; − 15.1)*	− 34.2 (− 51.9; − 16.4)*	− 48.3 (− 68.8; − 27.8)*	− 36.7 (− 56.5; − 16.9)*	Reference
Proportional IW (%)	− 1.88 (− 3.37; − 0.39)*	− 1.69 (− 3.05; − 0.32)*	0.19 (− 1.39; 1.77)	− 1.14 (− 2.66; 0.38)	Reference
IW (Watt)	− 0.34 (− 2.78; 2.11)	− 3.63 (− 5.87; − 1.39)*	− 0.34 (− 2.93; 2.25)	0.04 (− 2.45; 2.53)	Reference
Proportional IW (IW/Watt_max_ in %)	− 1.08 (− 4.01; 1.84)	− 2.79 (− 5.46; − 0.12)*	3.85 (0.76; 6.93)*	1.94 (− 1.05; 4.93)	Reference

Coefficients are derived from linear regression models adjust for age, sex, and body mass index.

CI confidence interval; *p < 0.05.

In addition, we analyzed differences in markers of spiroergometry between the patient groups in individuals with and without a reduced exercise capacity defined by a VO_2_@peak < 60% (Supplementary Tables 1, 2). In patients with reduced exercise capacity, we found a significantly higher VO_2_@rest and significantly lower IW (mL/min) and PIW in the CTEPH group compared to the patient controls. In the IPAH and the PAH-CTD group, VO_2_@rest values were significantly higher than in the patient controls and individuals in the IPAH group also had significantly lower VO_2_@peak values compared to the patient controls. In patients with preserved exercise capacity, the IPAH group had a significantly lower VO_2_@peak when compared with patient controls. The HFpEF group had significantly lower IW (ml/min and Watt) and PIW values than the patient controls.

In the whole patient population, the IW (ml/min and Watt) decreased significantly with age, while the PIW did not differ according to age (Table 4). Females had a significantly lower IW (ml/min and Watt) and a higher PIW than males. Adjusted for age and sex, the estimated systolic PAP, mean RAP, PAPmean, and RV/TLC were inversely associated with the IW, while BMI, obesity, TAPSE, FVC, FEV1/FVC, DLCOc, KCOc, maximal workload, heart rate at peak as well as VO_2_@VAT and VO_2_@peak were positively associated with the IW. The BMI and VO_2_@rest were positively associated with PIW, and the meanPAP showed an inverse associated with the PIW. RAPmean, PAPmean, and RV/TLC were inversely associated with IW (Watt). On the other hand, we observed positive associations of FVC, FEV1, maximal workload, and VO_2_@VAT with IW (Watt). TLC, diffusion capacity, maximal workload, VO_2_@rest, VO_2_@VAT and VO_2_@peak were associated with lower PIW (Watt) values.

**Table 4:** Association of metabolic and functional parameters with (proportional) internal work (IW) in the patients (n = 580).

	IW (ml/min)	Proportional IW	IW (Watt)	Proportional IW (Watt)
Age; years	− 1.32 (− 1.81; − 0.84)*	− 0.00 (− 0.04; 0.04)	− 0.16 (− 0.22; − 0.11)*	0.01 (− 0.07; 0.08)
Females versus males	− 20.3 (− 33.0; − 7.71)*	1.27 (0.35; 2.19)*	− 2.01 (− 3.37; − 0.64)*	1.63 (− 0.18; 3.45)
Smoking	− 3.16 (− 18.2; 11.9)	0.39 (− 0.70; 1.49)	0.29 (− 1.33; 1.91)	1.75 (− 0.41; 3.91)
Body mass index	3.90 (2.86; 4.95)*	0.08 (0.01; 0.16)*	0.11 (− 0.01; 0.23)	0.05 (− 0.11; 0.20)
Overweight	12.5 (− 3.10; 28.0)	− 0.34 (− 1.52; 0.83)	0.52 (− 1.22; 2.26)	− 0.18 (− 2.50; 2.14)
Obesity	46.5 (31.1; 61.8)*	0.78 (− 0.38; 1.92)	1.13 (− 0.59; 2.84)	− 0.08 (− 2.36; 2.21)
Type 2 diabetes	0.97 (− 14.1; 16.0)	0.28 (− 0.81; 1.38)	− 0.68 (− 2.31; 0.94)	0.15 (− 2.02; 2.31)
Hypertension	− 3.77 (− 17.3; 9.74)	− 0.48 (− 1.47; 0.51)	− 0.24 (− 1.70; 1.22)	− 0.94 (− 2.88; 1.00)
TAPSE	3.46 (2.16; 4.76)*	0.09 (− 0.01; 0.18)	0.13 (− 0.01; 0.28)	− 0.01 (− 0.22; 0.19)
Estimated systolic PAP	− 0.85 (− 1.19; − 0.51)*	− 0.03 (− 0.05; 0.01)	− 0.01 (− 0.05; 0.03)	0.04 (− 0.01; 0.10)
Mean right atrial pressure (mmHg)	− 2.22 (− 3.15; − 1.29)*	− 0.07 (− 0.14; 0.01)	− 0.15 (− 0.25; − 0.05)*	0.02 (− 0.12; 0.15)
PAPmean (mmHg)	− 1.60 (− 1.96; − 1.24)*	− 0.03 (− 0.06; − 0.01)*	− 0.04 (− 0.08; − 0.01)*	0.09 (0.04; 0.15)
PAWP (mmHg)	− 0.62 (− 1.50; 0.25)	− 0.02 (− 0.09; 0.04)	− 0.09 (− 0.19; 0.01)	− 0.03 (− 0.16; 0.10)
Total lung capacity (TLC) (% predicted)	0.11 (− 0.36; 0.58)	− 0.01 (− 0.05; 0.02)	− 0.01 (− 0.06; 0.04)	− 0.07 (− 0.14; − 0.01)*
Vital capacity (VC) (% predicted)	0.29 (− 0.10; 0.69)	− 0.00 (− 0.03; 0.03)	0.04 (− 0.01; 0.08)	− 0.04 (− 0.10; 0.02)
Forced vital capacity (FVC) (% predicted)	0.48 (0.09; 0.87)*	0.01 (− 0.02; 0.04)	0.05 (0.01; 0.09)*	− 0.04 (− 0.10; 0.02)
FEV1 (% predicted)	0.66 (0.28; 1.04)*	0.01 (− 0.02; 0.04)	0.06 (0.02; 0.10)*	− 0.03 (− 0.09; 0.03)
FEV1/FVC (%)	1.00 (0.28; 1.73)*	0.03 (− 0.02; 0.09)	0.05 (− 0.03; 0.13)	0.04 (− 0.08; 0.15)
Residual volume (RV) (% predicted)	− 0.13 (− 0.38; 0.11)	− 0.01 (− 0.03; 0.01)	− 0.02 (− 0.05; 0.01)	− 0.02 (− 0.06; 0.01)
RV/TLC (%)	− 1.04 (− 1.91; − 0.16)*	− 0.03 (− 0.10; 0.03)	− 0.16 (− 0.26; − 0.06)*	− 0.03 (− 0.16; 0.11)
DLCOc (% predicted)	1.17 (0.72; 1.62)*	− 0.02 (− 0.05; 0.02)	− 0.02 (− 0.08; 0.03)	− 0.15 (− 0.22; − 0.08)*
KCOc (% predicted)	0.94 (0.53; 1.36)*	− 0.01 (− 0.04; 0.02)	− 0.02 (− 0.07; 0.03)	− 0.13 (− 0.19; − 0.06)*
Maximal workload (Watt)	0.42 (0.29; 0.55)*	− 0.01 (− 0.02; 0.01)	0.02 (0.01; 0.04)*	− 0.05 (− 0.07; − 0.03)*
Maximal workload (% predicted)	0.15 (0.07; 0.24)*	− 0.00 (− 0.01; 0.01)	0.01 (− 0.01; 0.02)	− 0.02 (− 0.04; − 0.01)*
Heart rate (maximal)	0.55 (0.26; 0.84)*	− 0.00 (− 0.03; 0.02)	0.06 (0.03; 0.09)	− 0.03 (− 0.07; 0.01)
VO_2_ (ml/min) at rest	− 0.01 (− 0.10; 0.07)	− 0.016 (− 0.022; − 0.010)*	− 0.01 (− 0.02; 0.01)	− 0.013 (− 0.025; − 0.001)*
VO_2_ (ml/min) at VAT	0.17 (0.15; 0.20)*	0.001 (− 0.001; 0.003)	0.006 (0.003; 0.009)*	− 0.005 (− 0.009; − 0.001)*
VO_2_ (ml/min/kg) at peak	7.70 (6.35; 9.05)*	− 0.06 (− 0.17; 0.05)	0.24 (0.08; 0.40)	− 0.63 (− 0.85; − 0.43)*

Data are reported as β coefficients and 95%-confidence interval derived from linear regression models adjusted for age and sex; *p < 0.05.

In the HFpEF group maximal workload (Watt and % predicted) was positively associated with the IW (Watt) (Supplementary Table 3). We observed also a significant positively association of the PIW with PAPmean. The PIW (Watt) was inversely associated with lung function values (VC, FVC, FEV-1, DLCOc), maximal workload (Watt and % predicted), and VO_2_@VAT and VO_2_@peak.

## Discussion

In our study we showed that patients have a similar PIW compared to healthy individuals from the general population. In 580 patients as well as in 354 SHIP controls the PIW was between 14 and 15%, which correspondents well with the PIW of 14.7%, which we previously reported for the whole SHIP-cohort^14^. While the PIW did only differ slightly between the patients and the SHIP controls, the PIW (Watt) was significantly higher in the patients than in the SHIP controls (18.0% vs. 15.9%). Also the median IW was higher in patients than in the SHIP controls.

Over all patients groups, the IPAH patients had the highest PIW followed by the patient controls. A similar pattern was observed when using the aerobic capacity only up to the VAT for the calculation of the PIW (Watt). In the study by Shah et al. a median IW (Watt) of 32.4 and a median PIW (Watt) of 27% was reported for HFpEF patients^13^. These values were much higher compared to those found in our study, but may be explained by the different CPET protocol or a different disease severity of the patients. The HFpEF patients in our study were in median 10 years older and were functionally more restricted compared to those in the other study (RAPmean: 10 vs. 3 mmHg, PAPmean 32 vs. 18 mmHg; PCWP 19 vs. 7 mmHg, maximal workload 68 vs. 86 Watt).

While the median (P)IW (Watt) values differed substantially between the HFpEF patients in our study and those in the study from Shah et al., the median values of these parameters were comparable between the SHIP controls in our study and 3,047 individuals of the Framingham heart study (IW (Watt): 25.3 vs. 29.1; PIW (Watt): 15.9% vs. 15.0%)^13^. This indicates that the PIW may be a useful screening parameter in healthy individuals independent of the CPET protocol. In a previous analysis based on SHIP data, we demonstrated that a higher PIW was associated with a higher risk to die^14^. Future studies are needed to reveal, whether the PIW adds predictive value independent of established cardiovascular risk factors such as age, sex, BMI, blood pressure, diabetes or dyslipidaemia.

In our study we saw a difference in the PIW(Watt) between patients and controls but no such difference for the PIW. This finding suggest that the PIW(Watt) may be a useful clinical marker for differentiation between healthy and sick patients. The PIW, however, seems not discriminate well between healthy and sick persons in our analyses. Thus, further studies are needed to evaluate this parameter, which has not been considered in any previous patient study.

In our previous analysis of the population-based SHIP, we demonstrated that female sex, current smoking, BMI, and type 2 diabetes were associated with a higher PIW^14^. Likewise, a higher VO_2_@peak was associated with a lower PIW in a non-linear fashion^14^. In the patient population of the present analysis we observed positive associations of female sex and BMI with the PIW, but found no significant associations of current smoking, type 2 diabetes and VO_2_@peak with the PIW. Likewise, in 3,047 individuals of the Framingham Heart Study (FHS) female sex, BMI, and low VO_2_@peak were described as risk factors for a higher IW (Watt), while in the HFpEF patients only BMI and low VO_2_@peak were associated with a higher IW^13^. Thus, female sex, BMI and low VO_2_@peak seem to be major contributors for a higher (P)IW in healthy individuals, while in patients with pulmonary hypertension the distribution of potential risk factors may vary according to the disease pattern and the disease severity of the patients. Similarly, the association of the IW with the VO_2_@peak may get weaker with increasing disease severity.

A strength of our study is that we included five different patient groups allowing us to investigate the PIW in different disease patterns. Furthermore, the patient groups as well as the healthy SHIP controls followed both the same CPET protocol. The unloading cycling phase was only 1 min in our study, but 3 min in the study by Shah et al.^13^. However, previous data in patients with pulmonary hypertension^25^ and COPD^26^ demonstrated, that longer unloading cycling phases lead to a lower VO_2_@peak. Furthermore, our patients had a higher disease severity than those in the study of Shah et a. making the comparison between these two studies difficult.

The PIW was similar between patients and healthy controls, but after consideration of the aerobic efficiency, patients needed a higher amount of their maximal workload when changing from resting to unloading exercise. Such a difference was also observed, when considering only the submaximal workload up to the VAT. Since the PIW(Watt) was higher in patients with pulmonary hypertension, this marker may be a useful CPET parameter in clinical practice, because, in contrast to most of the currently used CPET parameters, the PIW does not request a maximal workload for the patient. Further studies are needed to validate the prognostic significance of the PIW.

### Supplementary Information

Supplementary Information.

## Data Availability

Data from the “Study of Health of Pomerania” are available from the University Medicine Greifswald, Germany but restrictions apply to the availability of these data, which were used under license for the current study, and so are not publicly available. Data are, however, available upon reasonable request at https://transfer.ship-med.uni-greifswald.de/FAIRequest/data-use-intro and with permission of the University Medicine Greifswald.
