# Supplementary material for: Metabolic cost of unloading pedalling in different groups of patients with pulmonary hypertension and volunteers
Source: Sci Rep. 2024 Mar 5;14:5394. doi: 10.1038/s41598-024-55980-z (PMC10915286; doi:10.1038/s41598-024-55980-z)
Supplement: Supplementary file 1 — Supplementary Information. [file 41598_2024_55980_MOESM1_ESM.docx]

**Supplementary Figure 1.** Flow chart of the study population

**
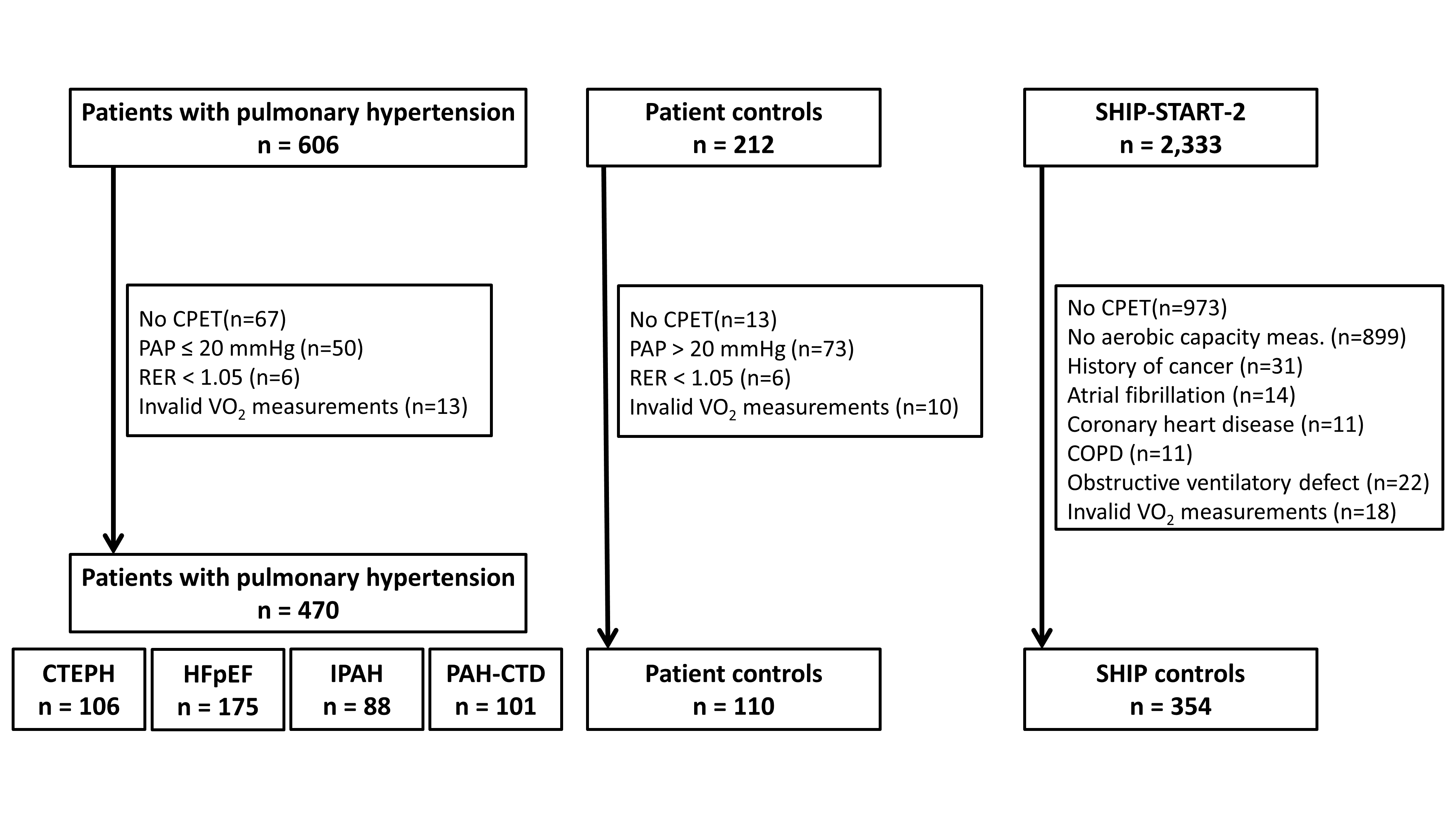
**

**Supplementary Table 1.** Associations of the groups with markers of spiroergometry compared to patient controls (VO2peak < 60 % [n=275])

|  | **CTEPH**  β (95%-CI) | **HFpEF**  β (95%-CI) | **IPAH**  β (95%-CI) | **PAH-CTD**  β (95%-CI) | **Patient controls**  β (95%-CI) |
| --- | --- | --- | --- | --- | --- |
| **VO_2_ (ml/min) at rest** | 45.5 (17.2; 73.7)* | 23.1 (-3.85; 50.1) | 28.5 (0.85; 56.1)* | 49.0 (20.6; 77.3)* | reference |
| **VO_2_ (ml/min) at unloading exercise** | 18.9 (-15.7; 53.5) | 10.0 (-22.9; 43.0) | 3.81 (-30.0; 37.6) | 25.7 (-8.99; 60.4) | reference |
| **VO_2_ (ml/min) at peak** | -35.0 (-111; 41.0) | -45.7 (-118; 26.8) | -104 (-179; -30.2)* | -114 (-190; 37.8) | reference |
| **Internal Work (IW) (ml/min)** | -26.6 (-52.4; -0.78)* | -13.1 (-37.7; 11.6) | -24.6 (-49.9; 0.56) | -23.3 (-49.1; 2.62) | reference |
| **Proportional IW (%)** | -2.95 (-5.43; -0.46)* | -1.38 (-3.75; 1.00) | -0.66 (-3.08; 1.77) | -1.25 (-3.75; 1.25) | reference |
| **IW (Watt)** | -1.02 (-4.73; 2.68) | -2.5 (-6.07; 0.97) | 1.15 (-2.46; 4.75) | 1.85 (-0.85; 6.55) | reference |
| **Proportional IW (IW/Watt_max_ in %)** | -3.46 (-8.84; 1.91) | -4.45 (-9.55; 0.66) | 1.37 (-3.86;; 6.60) | 3.03 (-2.37; 8.44) | reference |

Coefficients are derived from linear regression models adjusted for age, sex, and body mass index

CI confidence interval; * p<0.05

**Supplementary Table 2.** Associations of the groups with markers of spiroergometry compared to patient controls (VO2peak ≥ 60 % [n=305])

|  | **CTEPH**  β (95%-CI) | **HFpEF**  β (95%-CI) | **IPAH**  β (95%-CI) | **PAH-CTD**  β (95%-CI) | **Patient controls**  β (95%-CI) |
| --- | --- | --- | --- | --- | --- |
| **VO_2_ (ml/min) at rest** | 10.9 (-11.7; 33.5) | 19.4 (-0.51; 39.4) | -2.46 (-31.1; 26.1) | 14.3 (-9.27; 37.8) | reference |
| **VO_2_ (ml/min) at unloading exercise** | -10.1 (-39.8; 19.7) | -18.0 (-44.2; 8.18) | -20.0 (-57.6; 17.6) | -7.28 (-38.2; 23.7) | reference |
| **VO_2_ (ml/min) at peak** | -58.0 (-148; 32.1) | -69.7 (-149; 9.67) | -280 (-393; -166)* | -67.0 (-161; 26.7) | reference |
| **Internal Work (IW) (ml/min)** | -21.0 (-45.5; 3.64) | -37.5 (-59.1; -15.8)* | -17.6 (-48.6; 13.5) | -21.6 (-47.1; 4.02) | reference |
| **Proportional IW (%)** | -1.03 (-2.90; 0.85) | -2.10 (-3.75; -0.45)* | 1.39 (-0.98; 3.76) | -1.13 (-3.08; 0.82) | reference |
| **IW (Watt)** | -0.32 (-3.02; 2.39) | -3.52 (-5.90; -1.13)* | 0.00 (-3.42; 3.42) | -1.59 (-4.41; 1.22) | reference |
| **Proportional IW (IW/Watt_max_ in %)** | -0.14 (-3.19; 2.91) | 2.34 (-5.03; 0.34) | 3.68 (-0.18; 7.53) | -0.92 (-4.09; 2.25) | reference |

Coefficients are derived from linear regression models adjusted for age, sex, and body mass index

CI confidence interval; * p<0.05

**Supplementary Table 3.** Associations of internal work (IW) and proportional internal work (in %) with functional parameters in the HFpEF group

|  | **IW (Watt)** | **PIW (Watt)** |
| --- | --- | --- |
| TAPSE | 0.04 (-0.06; 0.15) | 0.01 (-0.06; 0.08) |
| Estimated systolic PAP | 0.25 (-0.12; 0.62) | 0.18 (-0.05; 0.41) |
| Mean right atrial pressure (mmHg) | -0.07 (-0.20; 0.07) | 0.05 (-0.04; 0.15) |
| PAPmean (mmHg) | 0.04 (-0.17; 0.24) | 0.20 (0.07; 0.33)* |
| PAWP (mmHg) | -0.00 (-0.13; 0.13) | 0.08 (-0.01; 0.17) |
| Total lung capacity (TLC) (% predicted) | -0.01 (-0.41; 0.39) | -0.26 (-0.54; 0.01) |
| Vital capacity (VC) (% predicted) | -0.01 (-0.46; 0.44) | -0.38 (-0.69; -0.07)* |
| Forced vital capacity (FVC) (% predicted) | -0.01 (-0.46; 0.43) | -0.38 (-0.69; -0.08)* |
| FEV1 (% predicted) | -0.09 (-0.55; 0.38) | -0.46 (-0.77; -0.15)* |
| FEV1/FVC (%) | -0.01 (-0.25; 0.23) | -0.11 (-0.27; 0.06) |
| Residual volume (RV) (% predicted) | -0.16 (-0.89; 0.58) | -0.22 (-0.74; 0.30) |
| RV/TLC (%) | -0.03 (-0.25; 0.19) | 0.13 (-0.03; 0.28) |
| DLCOc (% predicted) | -0.13 (-0.62; 0.37) | -0.45 (-0.79; -0.11)* |
| KCOc (% predicted) | 0.02 (-0.54; 0.57) | -0.31 (-0.71; 0.08) |
| Maximal workload (Watt) | 0.71 (0.33; 1.09)* | -0.48 (-0.74; -0.23)* |
| Maximal workload (% predicted) | 0.50 (0.13; 0.88)* | -0.48 (-0.73; -0.24)* |
| Heart rate (maximal) | 0.30 (-0.08; 0.69) | -0.10 (-0.37; 0.16) |
| VO_2_ (ml/min) at rest | -1.15 (-2.42; 0.11) | -0.40 (-1.25; 0.45) |
| VO_2_ (ml/min) at VAT | -0.98 (-5.09; 3.13) | -2.92 (-5.66; -0.19)* |
| VO_2_ (ml/min/kg) at peak | 0.01 (-0.05; 0.06) | -0.08 (-0.11; -0.04)* |

Data are reported as β coefficients and 95%-confidence interval derived from linear regression models adjusted for age and sex; *p<0.05
